# Use of a standardized diagnostic approach improves the prognostic information of histopathologic factors in pancreatic and periampullary adenocarcinoma

**DOI:** 10.1186/1746-1596-9-80

**Published:** 2014-04-14

**Authors:** Jacob Elebro, Karin Jirström

**Affiliations:** 1 Department of Clinical Sciences, Division of Oncology and Pathology, Lund University, Skåne University Hospital, Lund SE-221 85, Sweden; 2 Skåne University and Regional Laboratories, Lund, Sweden

**Keywords:** Carcinoma, Pancreatic ductal, Common bile duct neoplasms, Duodenal neoplasms, Pathology, Surgical, Pancreaticoduodenectomy, Prognosis

## Abstract

**Background:**

Variability in reported histopathology parameters in operated periampullary adenocarcinomas may affect the prognostic weight of the parameters. Standardized axial sectioning produces a higher incidence of involved margins and also seems to produce a lower relative incidence of pancreatic compared with distal bile duct origin and a higher incidence of involved lymph nodes, compared with non-standardized procedure. The aims of this study were to 1) assess how a previously not described standardized pathology procedure, with longitudinal sectioning along the distal bile duct, affects reported tumour origin, margin status and involved lymph nodes, compared with non-standardized procedure, 2) assess if re-evaluation of microscopic slides affects the prognostic value of margin status and 3) compare the results of this standardized procedure with reported results of other standardized and non-standardized procedures.

**Methods:**

One hundred seventy-five consecutive pancreaticoduodenectomy specimens with primary adenocarcinomas, operated during 2001 – 2011 at the University hospitals of Lund and Malmö, Sweden, were re-evaluated histologically, and parameters relevant for classification and prognosis were assessed, with 1 mm as a threshold for involved or uninvolved margins. Follow-up lasted until 31 December 2013. Five-year overall survival (OS) and hazard ratios (HR) were calculated for the margin status stated in the original reports and margin status after re-evaluation.

**Results:**

Compared with non-standardized cases (n = 129), standardized cases (n = 46) had more involved lymph nodes in the specimens (median 3 vs 1), a higher fraction of distal bile duct origin (39% vs 21%) and a higher fraction of involved margins (74% vs 47%). The prognostic value of uninvolved margins increased by re-evaluation of slides (p < 0.001) and the adjusted HR for involved margins increased from 1.6 (95% CI 1.1 - 2.4) to 3.3 (95% CI 1.5 – 7.0). Uninvolved margins remained a significant predictor of OS in adjusted analysis.

**Conclusions:**

Both the method of sectioning the specimen and the microscopic assessment affect prognostic pathology parameters significantly. The results of the herein described standardized method are similar to the results of other standardized procedures. The 1-mm threshold for involved margins in pancreaticoduodenectomies is relevant for OS, and margin status is an independent prognostic parameter.

**Virtual slides:**

The virtual slides for this article can be found here: http://www.diagnosticpathology.diagnomx.eu/vs/1056639379120615

## Background

Pathology guidelines that change the incidence of histopathology parameters are clinically relevant since the parameters carry prognostic information. Guidelines on gross examination and sectioning of pancreaticoduodenectomy (PD) specimens have changed during the last years, after the introduction of the Leeds pathology protocol (LEEPP) [1]. This standardized procedure raised the incidence of involved margins (R1) and involved lymph nodes (N1), and also decreased pancreatic origin and increased distal bile duct origin [2,3] compared to large series using non-standardized procedures [4-10].

Proportions of tumour origin vary greatly between different series of operated periampullary adenocarcinomas and it is not known which proportions most accurately reflect the biology of the tumours, or are most clinically relevant. It is however evident that a meticulous pathology examination improves the quality of the pathology report for these cancer forms by producing a higher incidence of N1 and R1 [2]. A high proportion of R1 also seems to correlate to a low relative incidence of pancreatic origin, suggesting that a more thorough examination decreases the relative incidence of pancreatic origin [11]. So far, the reported increase of R1 and decrease in pancreatic origin in the LEEPP-series has been attributed to this particular slicing method. It is however not clear to what extent this change is due to the method or to the interest and dedication of the pathologist.

Here, we present the results of a different standardized protocol (SP), in which the pathologist gains access to the full length of the common bile duct through a longitudinal opening via the posterior margin of the PD-specimen, and only standard size blocks are made. It has been stated that this method is inferior to the LEEPP, due to its limited value for assessing tumour origin and resection margins [1]. This method has however not been studied in a standardized setting before.

## Methods

### Data collection and patient characteristics

The study cohort is a retrospective consecutive series of 175 PD-specimens with primary adenocarcinomas surgically treated at the University hospitals of Lund and Malmö, Sweden, from January 1 2001 until December 31 2011. Data on survival were gathered from the Swedish National Civil Register. Follow-up started at the date of surgery and ended at death or at December 31 2013, whichever came first.

Data on margin status was collected from the original pathology reports, as were data on age at surgery, date of surgery, sex, and whether the specimen was handled according to the SP or not. Data was also gathered on the origin of lymph nodes submitted in separate containers. After information was given on how and from where the surgeons harvested lymph nodes submitted in separate containers, positions 6, 8, 12, 13, 14 and 17 were classified as originating from the specimen, and other positions including 9 and 16 were classified as not originating from the specimen.

Of the 175 PDs, 46 (26%) were examined and sectioned according to our SP by one pathologist (JE) and 129 (74%) were examined and sectioned by several pathologists according to personal choice (non-standardized protocol, NSP).

Ethical permission was obtained from the Ethics Committee at Lund University.

### Sectioning of the specimens, standardized protocol

This method is, by opening the PD-specimen along the bile duct, similar to one of the methods earlier described by the Royal College of Pathologists [12] but performed in a standardized manner and without opening the pancreatic duct.

**Figure 1:**
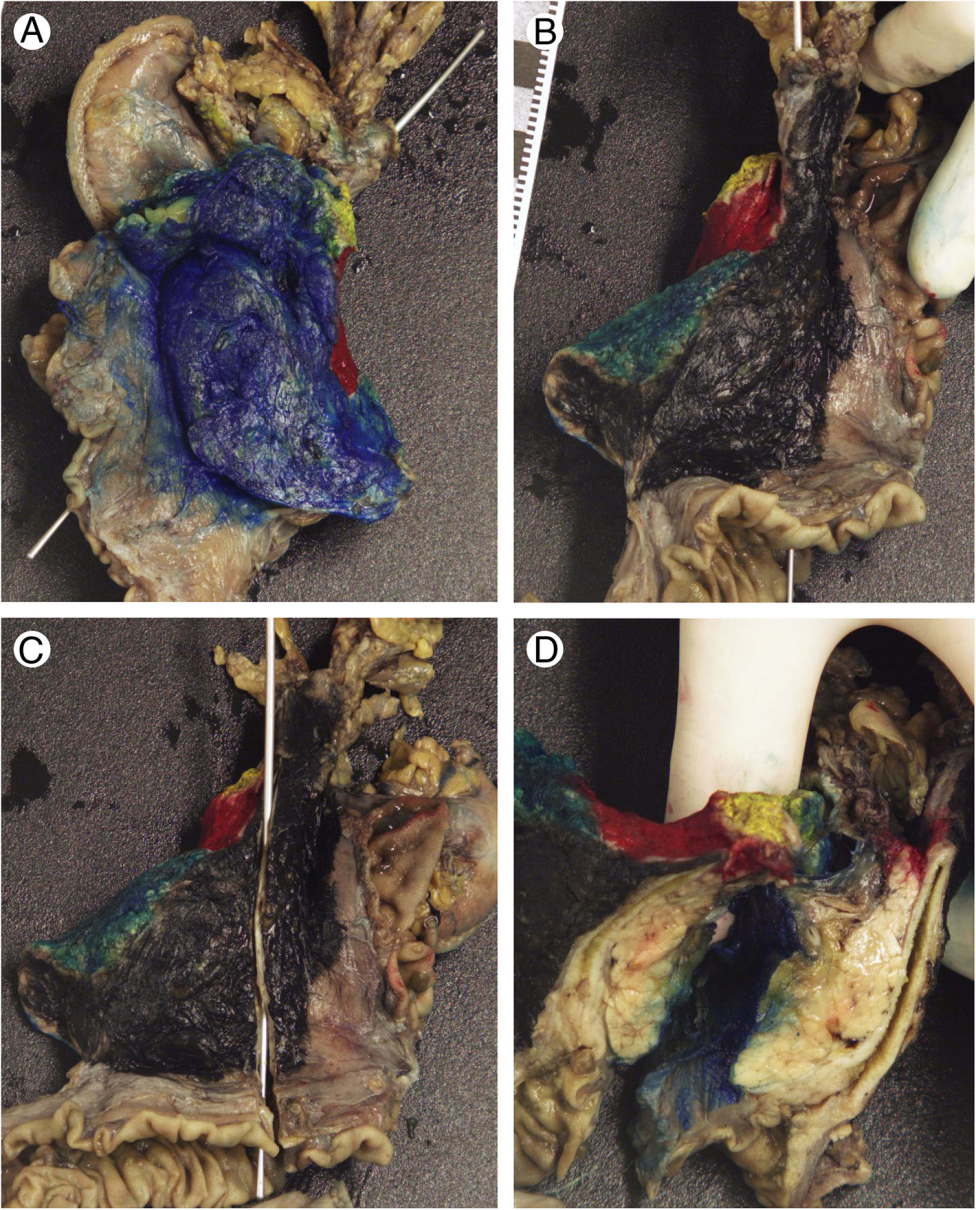
**Accessing a pancreaticoduodenectomy specimen through the bile duct. (A)** Anterior view of a painted pancreaticoduodenectomy specimen. Posterior – black, anterior – blue, pancreatic transection margin – yellow, SMV-margin - red and SMA-margin - green. A probe is inserted in the lumen of the bile duct. **(B)** posterior view, **(C)** posterior view with the bile duct opened longitudinally, and **(D)** the pancreatic parenchyme accessed through the bile duct, visualizing the ampulla, the bile duct and parts of the pancreatic parenchyme, as well as parts of the margins.

The specimens were handled after fixation in formalin (Figure 1). Margins were stained in different colours; one for the pancreatic transection margin, one for the margin towards the superior mesenteric vein (SMV), one for the margin towards the superior mesenteric artery (SMA), one for the anterior surface and one for the posterior margin. The specimens were accessed through a longitudinal opening of the common bile duct at the posterior margin, from the most proximal part of the bile duct through the papilla of Vater. In the same plane the section was deepened through the common bile duct and into the pancreatic parenchyme. This produced a book-like opening that visualized the whole length of the common bile duct, the ampulla and adjacent pancreatic parenchyme as well as parts of the posterior margin and parts of the SMV-margin. Several standard size blocks were sampled from the ampulla with adjacent duodenal mucosa, pancreatic parenchyme and anterior and posterior margins. The bile duct was sampled longitudinally, with adjacent pancreatic parenchyme, posterior margin and SMV-margin. Additional standard size blocks were sampled from the SMA-margin, from all visible or palpable lymph nodes in the specimen and from additional areas with possible tumour growth. En face sections were made from the pancreatic, bile duct, pyloric and duodenal transection margins.

### Standardized protocol vs non-standardized protocol

#### Re-evaluations of slides

All haematoxylin & eosin stained slides from all cases were revised by one pathologist (JE), blinded to the original report and outcome. Other stains were not revised or used for the assessment of any parameter. Data were gathered on tumour origin, size and grade, perineural invasion, lymphatic vessel and blood vessel invasion, invasion of peripancreatic fat, number of lymph nodes and involved lymph nodes found by the pathologist in the specimen, number of lymph nodes and involved lymph nodes harvested from the specimen by the surgeon and submitted in separate containers, number of lymph nodes and involved lymph nodes in separate containers originating from other areas, N-stage, T-stage and margin status.

Decision on tumour origin was based on the anatomical centre of the tumour, with the aid of preinvasive precursor lesions or multifocality, if present. A tumour in the duodenal mucosa with intestinal morphology that involved the ampulla in the periphery was considered to be of duodenal origin. A similar tumour with the ampulla in the centre was considered to be of ampullary origin. A tumour along the bile duct that involved the ampulla was considered to be of bile duct origin if the ampulla was in the periphery of the tumour, but of ampullary origin if the ampulla was in the centre. Multifocal tumour growth or multifocal premalignant changes in the pancreatic parenchyme in the absence of evidence of other tumour origin was considered as a sign of pancreatic origin. In addition to tumour origin the distinction between intestinal morphology and pancreaticobiliary morphology was made for all ampullary carcinomas using morphological criteria [13].

For the assessment of tumour grade, only the poorest degree of differentiation was recorded.

Margin status was denoted as R1 if cancer was present less than 1 mm from any margin except for the duodenal serosa, as R0 if the shortest distance exceeded 1 mm, and as unknown (Rx) if any margin, except the duodenal serosa close to the cancer, was insufficiently sampled. If a margin was considered sufficiently sampled or not differed by the location of the tumour. In addition to pancreatic and distal bile duct transection margins, an ampullary carcinoma needed at least one standard size block showing the relation to the anterior surface, adjacent to the duodenal wall, two showing the relation to the posterior surface adjacent to the duodenal wall, one from the SMA-margin and one from the SMV-margin, in order to be considered sufficiently sampled regarding margins. Carcinomas of pancreatic or distal bile duct origin needed, in addition to pancreatic and distal bile duct transection margins, at least two blocks showing the relation to the posterior margin, one from the SMA-margin, one from the SMV-margin and one from the anterior margin. For duodenal origin, one block each from the posterior and anterior margins adjacent to the duodenal wall was considered sufficient. A case could be considered as R1 in an unspecified margin even if other margins were insufficiently sampled.

For sampling of lymph nodes in the specimen, the full surface around the specimens was searched manually and also visually after sectioning in intervals of approximately 3 mm.

#### Statistical analysis

The Chi-square test and Fisher’s Exact test were used to analyse differences in the distribution of histopathological factors in relation to use of standardized vs non-standardized protocol, and according to tumour location. Kaplan-Meier analysis and log rank test were used to illustrate differences in 5-year overall survival (OS) in strata according to margin status. Cox regression models were used to calculate hazard ratios (HR) for the impact of histopathology parameters on 5-year OS, in univariable and multivariable analysis, adjusted for age, sex, tumour morphology, tumour size, tumour grade, T-stage, N-stage, margin status, perineural invasion, growth in peripancreatic fat, invasion of lymphatic vessels and invasion of blood vessels. Cases who died within 1 month from surgery (n = 2) or were lost to follow up (n = 1) were excluded from the survival analyses.

All tests were two-sided and a p-value <0.05 was considered statistically significant. All statistical analyses were performed using IBM SPSS Statistics version 20.0 (SPSS Inc., Chicago, IL, USA).

## Results

The annual PD-rate increased during the study period, with 35 and 29 cases operated in 2010 and 2011, respectively, compared to a median of 13 per year (range 8–19) during 2001–2009. Forty-two of the 46 SP-cases were diagnosed during 2010 – 2011, which coincided with an increased number of lymph nodes sent for analysis in separate containers; median 1 (interquartile range, IQR 0 – 2) during 2001 – 2009 and median 7 (IQR 3.25 – 10) during 2010 – 2011.

Median 5-year OS was 30.4 months in the full cohort of all 172 SP- and NSP-cases, 35.0 months in the SP-group and 29.7 months in the NSP-group. In the SP-group of 46 cases, 27 died during follow up and 19 were censored at December 31 2013. Out of the 129 NSP-cases, 3 were excluded from the survival analysis, but included in all other analyses. Of the remaining 126 cases, 88 died during follow up and 38 were censored at December 31 2013.

### Differences in the distribution of histopathological parameters between SP-cases and NSP-cases

As shown in Table 1, there were several significant differences in the distribution of histopathological parameters between the re-evaluated NSP- and SP-materials.

**Table 1:** Standardized vs non-standardized protocol: Characteristics of 175 re-evaluated periampullary adenocarcinomas

	**NSP**	**SP**	**p-value**	**All**
	**(n = 129)**	**(n = 46)**		**(n = 175)**
Tumour origin			**0.040**	
Duodenum	9 (7%)	5 (11%)		14 (8%)
Ampulla, both types	58 (45%)	12 (26%)		70 (40%)
Distal Bile Duct	27 (21%)	18 (39%)		45 (26%)
Pancreas	35 (27%)	11 (24%)		46 (26%)
Tumour size, mm				
M (IQR)	30 (20–35)	30 (25–40)	0.649	30 (21–35)
Larger than 20 mm	92 (71%)	42 (91%)	**0.008**	134 (77%)
Differentiation, poor	70 (54%)	31 (67%)	0.164	101 (58%)
Lymph nodes				
In PD specimen, M (IQR)	6 (3–10)	9 (7–13)	0.102	7 (4–10)
From PD specimens, M (IQR)	1 (0–2)	7 (3–9)	**<0.001**	2 (0–5)
Total PD specimen, M (IQR)	8 (5–12)	16 (12–19)	**<0.001**	11 (6–15)
≥10 lymph nodes PD specimen, n (%)	58 (45%)	40 (87%)	**<0.001**	98 (56%)
Other local lymph nodes, M (IQR)	0 (0–1)	0 (0–1)	0.400	0 (0–1)
Total, all lymph nodes, M (IQR)	9 (5–13)	16 (13–20)	**<0.001**	11 (6–16)
Involved lymph nodes				
In PD specimen, M (IQR)	1 (0–2)	3 (0–4)	**0.001**	1 (0–3)
From PD specimen, M (IQR)	0 (0–0)	0 (0–1)	**0.024**	0 (0–0)
Total PD specimen, M (IQR)	1 (0–2)	3 (0–4)	**0.023**	1 (0–3)
Other local, M (IQR)	0 (0–0)	0 (0–0)	**0.017**	0 (0–0)
Total, all involved lymph nodes, M (IQR)	1 (0–2)	3 (0–4)	**0.015**	1 (0–3)
N-stage, pN1 (for duodenum pN1-N2)	74 (57%)	33 (72%)	0.113	107 (61%)
Margin involvement			**<0.001**	
R1	60 (47%)	34 (74%)		94 (54%)
Rx (uncertain/unassessable)	56 (43%)	0 (0%)		56 (32%)
R0	13 (10%)	12 (26%)		25 (14%)
Perineural infiltration	71 (55%)	34 (74%)	**0.035**	105 (60%)
Infiltration in lymph vessels	79 (61%)	32 (70%)	0.374	111 (63%)
Infiltration in blood vessels	38 (29%)	4 (9%)	**0.004**	42 (24%)
Infiltration in peripancreatic fat	71 (55%)	36 (78%)	**0.008**	107 (61%)
T-stage			0.074	
pT1	8 (6%)	0 (0%)		8 (4%)
pT2	21 (17%)	3 (6%)		24 (14%)
pT3	70 (54%)	33 (72%)		103 (59%)
pT4	30 (23%)	10 (22%)		40 (23%)
Blocks from PD-specimen				
Regular blocks, Median (IQR)	15 (12–22)	23 (20–27)	**0.001**	
Mean (min - max)	17 (6–48)	24 (14–36)		
Large blocks, Median (IQR)	0 (0–2)	0 (0–0)	**<0.001**	
Mean (min - max)	1 (0–8)	0 (0–0)		

SP, standardized procedure. NSP, non-standardized procedure. PD, pancreatoduodenectomy. M, median. IQR, interquartile range. SMA, superior mesenteric artery. SMV, superior mesenteric vein. For margin involvement p-values were calculated R1 vs R0 and Rx. Bold text indicates p < 0.05.

Tumour origin differed between the SP-group and the NSP-group (p = 0.040), with a higher proportion of distal bile duct origin (39% vs 21%) and a lower proportion of ampullary origin (26% vs 45%) in the former.

There was no significant difference between the SP-group and the NSP-group regarding the number of lymph nodes found by the pathologist in the PD-specimens, but the number of lymph nodes harvested from the specimen by the surgeon, as well as the total number of lymph nodes originating from the PD-specimens, was significantly higher in the SP-group compared with the NSP-group (p < 0.001 for both).

The number of involved lymph nodes in the PD-specimens was also significantly higher in the SP-group as compared with the NSP-group (p = 0.001), and the number of involved lymph nodes from the PD-specimens submitted in separate containers and total number of involved lymph nodes originating from the specimens differed significantly. The proportion of cases with involved lymph nodes (N1-N2) did not differ significantly between the SP-group and NSP-group.

Since the increase in the number of lymph nodes harvested from the specimen by the surgeon occurred in 2009, a separate analysis on lymph node-variables was performed for the last 2.5 years of the study period (July 2009 – 2011). This revealed a significant difference between the SP-group (n = 44) and the NSP-group (n = 31) in the number of involved lymph nodes found in the PD-specimens by the pathologist (median 2.5 vs 1, p = 0.046). There were however no significant differences in the total number of lymph nodes from the specimen (median 16 vs 12, p = 0.601), fraction of cases with 10 or more lymph nodes (89% vs 74%, p = 0.128) or fraction of cases with involved lymph nodes (71% vs 65%, p = 0.622).

As further shown in Table 1, there was a significantly larger proportion of R1 cases (p = 0.002), tumours larger than 20 mm (p = 0.008), perineural tumour growth (p = 0.035) and infiltration of peripancreatic fat (p = 0.002) in the SP-group compared with the NSP-group. In contrast, infiltration of blood vessels was more often found in the NSP-group (p = 0.004).

We also examined the involvement of different resection margins by tumour type (Table 2). Significant differences (R0 vs R1 and Rx) between the SP and non-SP groups were found at the posterior margin (p = 0.001), the SMA-margin (p < 0.001) and the SMV-margin (p < 0.001), and in tumours of distal bile duct origin (p = 0.006).

**Table 2:** Margin status and tumour origin

	**Duodenum**		**Ampulla**		**Distal Bile Duct**		**Pancreas**		**All tumour origins**		
	**NSP**	**SP**	**NSP**	**SP**	**NSP**	**SP**	**NSP**	**SP**	**NSP**	**SP**	**p-value**
	**9**	**5**	**58**	**12**	**27**	**18**	**35**	**11**	**129**	**46**	
R1, n (%)	1 (11%)	2 (40%)	19 (33%)	5 (42%)	15 (56%)	17 (94%)	25 (71%)	10 (91%)	60 (47%)	34 (74%)	**0.002**
R0, n (%)	2 (22%)	3 (60%)	7 (12%)	7 (58%)	3 (11%)	1 (6%)	1 (3%)	1 (9%)	13 (10%)	12 (26%)	
Rx, n (%)	6 (67%)	0	32 (55%)	0	9 (33%)	0	9 (26%)	0	56 (43%)	0	
Pancreas transection margin	0	1	2	0	3	2	9	1	14 (11%)	4 (9%)	0.784
DBD transection margin	0	0	0	0	1	1	1	0	2 (2%)	1 (2%)	1.000
SMA margin	0	0	0	0	2	8	0	2	2 (2%)	10 (22%)	**<0.001**
Posterior surface	0	2	8	4	7	10	7	4	22 (17%)	20 (44%)	**0.001**
SMV surface	0	1	0	0	3	10	10	8	13 (10%)	19 (41%)	**<0.001**
Anterior surface	0	1	1	2	4	1	3	2	8 (6%)	6 (13%)	0.202

Margin status in 175 re-evaluated pancreaticoduodenectomies. NSP, non-standardized protocol. SP, standardized protocol. DBD, distal bile duct. SMA, superior mesenteric artery. SMV, superior mesenteric vein. For percentages and significances, calculations were made R0 vs R1 and Rx. In separate tumour origins, differences in R1-fraction between the SP-group and the NSP-group were significant in distal bile duct origin, (p = 0.006). Some NSP-cases were classified as R1 in an unspecified margin. R1-cases could have more than one involved margin. Bold text indicates p < 0.05.

### Effect of re-evaluations of slides

The distribution of histopathological characteristics in the total re-evaluated material, stratified by tumour origin, is shown in Table 3. In the original reports there were 14 NSP-cases without information on margin status. Re-evaluation of slides changed margin status for the NSP-group, increasing R1 from 45/115 to 60/129 and decreasing R0 from 70/115 to 12/129 (p < 0.001), and re-evaluations also rendered 56 NSP-cases with unknown margin status (Rx). Re-evaluation of slides rendered a non-significant increase of R1 in the SP-material, from 63% (29/46) to 76% (35/46) (p = 0.257).

**Table 3:** Distribution of clinicopathological characteristics according to tumour origin in 175 re-evaluated periampullary adenocarcinomas

	**Duodenum**	**Ampulla Intestinal type**	**Ampulla Pancreatobiliary type**	**Distal Bile Duct**	**Pancreas**	**All**
	**n = 14**	**n = 51**	**n = 19**	**n = 45**	**n = 46**	**n = 175**
Standardized Procedure	5 (36%)	7 (14%)	5 (26%)	18 (40%)	11 (24%)	46 (26%)
Age at surgery, M (IQR)	68 (62 – 74)	67 (59 – 70)	69 (62 – 75)	64 (59 – 71)	68 (62 – 72)	67 (61 – 72)
Gender, Female	6 (43%)	29 (57%)	9 (47%)	21 (47%)	21 (46%)	86 (49%)
Tumour size, mm, M (IQR)	40 (30 – 53)	23 (15–30)	30 (24 – 40)	26 (22 – 35)	30 (25 – 35)	30 (21 – 35)
Larger than 20 mm	13 (93%)	27 (53%)	18 (95%)	37 (82%)	39 (85%)	134 (77%)
High grade	7 (50%)	26 (51%)	9 (47%)	31 (69%)	28 (61%)	101 (58%)
Lymph nodes, M (IQR)	10 (6 – 13.5)	9 (6 – 16)	10 (5 – 17)	12 (8 – 16.5)	11.5 (6.75 – 16)	11 (6 – 16)
10 or more lymph nodes	8 (57%)	25 (49%)	10 (53%)	30 (67%)	27 (59%)	100 (57%)
Involved lymph nodes, M (IQR)	0 (0 – 2)	0 (0 – 2)	2 (1 – 7)	1 (0 – 4)	2 (0 – 3)	1 (0 – 3)
pN1	4 (29%)	24 (47%)	16 (84%)	27 (60%)	34 (74%)	106 (61%)
pN2	2 (14%)					
Perineural infiltration	4 (29%)	16 (31%)	14 (74%)	37 (82%)	34 (74%)	105 (60%)
Infiltration in lymph vessels	2 (14%)	34 (67%)	15 (79%)	33 (73%)	27 (59%)	111 (63%)
Infiltration in blood vessels	0 (0.0%)	5 (10%)	8 (42%)	14 (31%)	15 (33%)	42 (24%)
Infiltration in peripancreatic fat	6 (43%)	16 (31%)	17 (89%)	36 (80%)	32 (70%)	107 (61%)
T-stage (pTNM)						
T1	0	5 (10%)	0	1 (2%)	2 (4%)	
T2	1 (7%)	11 (22%)	0	2 (4%)	10 (22%)	
T3	6 (43%)	19 (37%)	2 (11%)	42 (93%)	34 (74%)	
T4	7 (50%)	16 (31%)	17 (89%)	0	0	
R1	3 (38%)	10 (43%)	14 (93%)	32 (89%)	35 (95%)	94 (79%)
R0	5 (63%)	13 (57%)	1 (7%)	4 (11%)	2 (6%)	25 (21%)
Rx,uncertain margin status (n)	6	28	4	9	9	56
Pancreatic transection margin	1 (13%)	1 (4%)	1 (7%)	5 (14%)	10 (27%)	18 (15%)
DBD transection margin	0	0	0	2 (6%)	1 (3%)	3 (3%)
SMA-margin	0	0	0	10 (28%)	2 (5%)	12 (10%)
Posterior margin	2 (25%)	6 (26%)	6 (40%)	17 (47%)	11 (30%)	42 (35%)
SMV-margin	1 (13%)	0	0	13 (36%)	18 (49%)	32 (27%)
Anterior margin	1 (13%)	1 (4%)	2 (13%)	5 (14%)	5 (14%)	14 (12%)
5-year OS, M (IQR), months	n.r. (37 - n.r.)	53 (26 - n.r.)	26 (15–40)	25 (16 - n.r.)	25 (13–42)	30 (17-n.r.)

M, median. IQR, interquartile range. OS, overall survival. N.r., not reached.

Re-evaluations revealed lymph node involvement in 20% (14/70) of NSP-cases that were N0 in the original report. This caused a non-significant change in fraction with involved lymph nodes in the NSP-group, from 46% (59/129) to 57% (73/129) (p = 0.105). Re-evaluations rendered no alterations in the fraction of involved lymph nodes in the SP-material.

### Overall survival in relation to margin status

**Figure 2:**
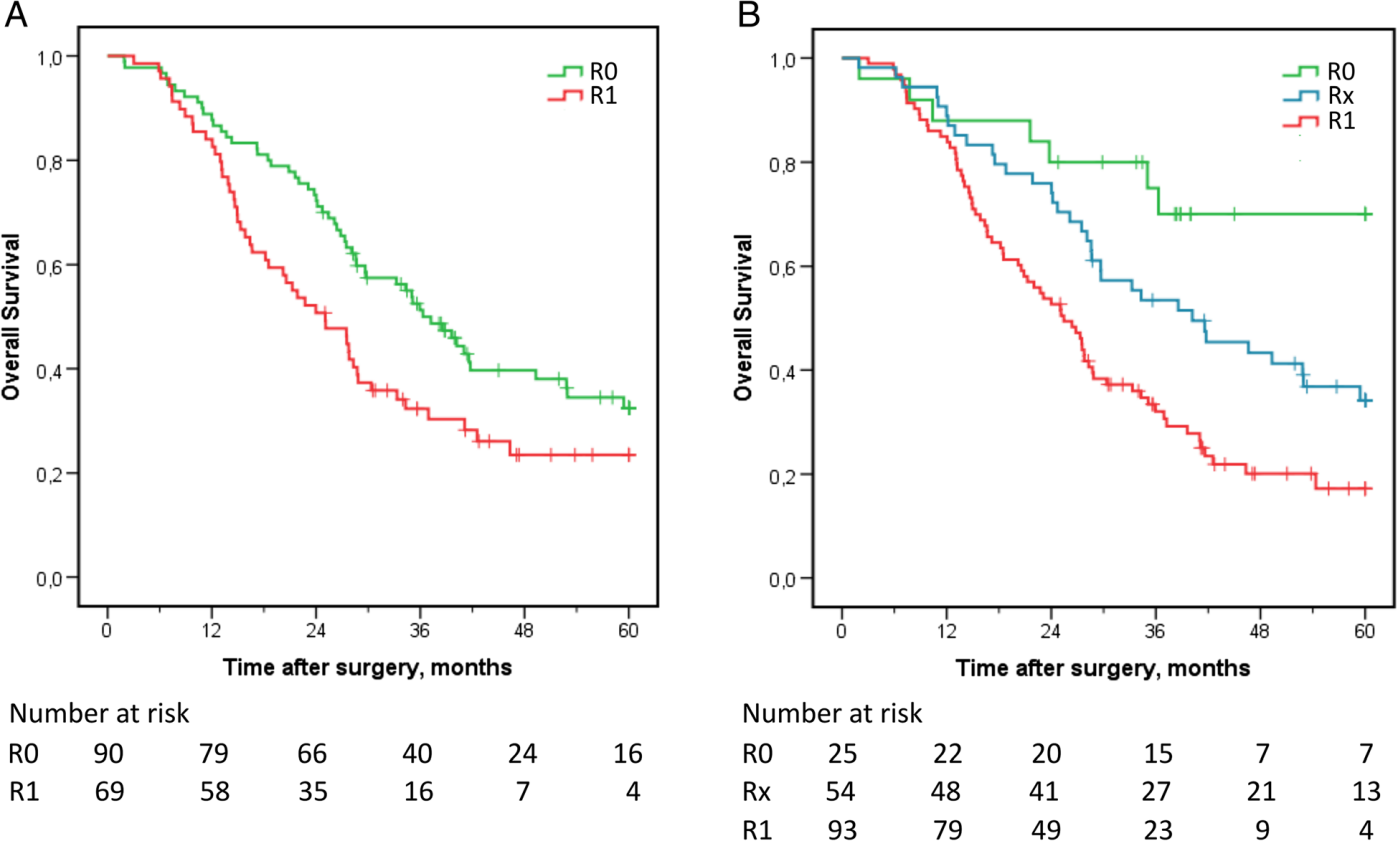
**Kaplan-Meier estimates of five-year overall survival in relation to margin status before and after re-evaluation.** Five-year OS in relation to **(A)** margin status stated in the original reports and **(B)** margin status upon re-evaluation of slides.

Kaplan-Meier analysis revealed a significantly prolonged five-year OS in the re-evaluated R0-group compared with the original report R0-group (p < 0.001) (Figure 2). As further shown in Table 4, the unadjusted HR for R1 vs R0 in the original report was 1.6 (95% CI 1.1 - 2.4). In the re-evaluated material the unadjusted HR for R1 vs R0 was 3.3 (95% CI 1.5 - 7.0) and the unadjusted HR for Rx vs R0 was 2.3 (95% CI 1.0 - 5.2). Re-evaluated, but not originally reported, margin status remained an independent prognostic factor in adjusted analysis (HR 2.2, 95% CI 1.0 - 4.9 for R1 and Rx vs R0) (Table 4). The unadjusted and adjusted HRs for re-evaluated histopathology parameters are shown in Table 5.

**Table 4:** Five year overall survival in relation to margin status in the original reports and after re-evaluation

	**Margin status in original reports**				**Margin status after re-evaluation**			
	**Number**	**Median OS (IQR)**	**Unadjusted HR (95% CI)**	**Adjusted HR (95% CI)**	**Number**	**Median OS (IQR)**	**Unadjusted HR (95% CI)**	**Adjusted HR (95% CI)**
R0	90	36.3 (23.1 - n.r.)			25	n.r. (35.0 - n.r.)		
Rx					54	40.2 (24.0 - n.r.)	2.3 (1.0 - 5.2)	2.2 (1.0 - 4.9)
R1	69	25.1 (14.0 - 46.3)	1.6 (1.1 - 2.4)	1.0 (0.7 - 1.6)	93	25.4 (14.6 - 41.6)	3.3 (1.5 - 7.0)	

Hazard ratios (HR) for risk of death within 5 years in relation to margin status, with R0 as reference. OS in the re-evaluated R0-group was significantly better than in the original report R0-group (p < 0.001). Differences in OS between the three levels of margin status in the re-evaluated material were significant; R0 vs R1 (p < 0.001), R1 vs Rx (p = 0.005) and R0 vs Rx (p = 0.043). HR for both original report and re-evaluated margin status adjusted for re-evaluated parameters.

R0, uninvolved margins. Rx, uncertain margin status. R1, cancer less than 1 mm from margin. IQR, interquartile range. CI, confidence interval. N.r., not reached.

**Table 5:** Unadjusted and adjusted hazard ratios for death within 5 years in relation to re-evaluated histopathology parameters

	**n (events)**	**Unadjusted HR (95% CI)**	**p-value**	**Adjusted HR (95% CI)**	**p-value**
**Age, continuous**	172 (112)	1.0 (1.0-1.0)	0.479	1.0 (1.0-1.1)	**0.015**
**Sex**					
Female	86 (47)				
Male	86 (65)	1.5 (1.0-2.2)	**0.042**	1.4 (0.9-2.0)	0.131
**T-stage**					
T1	8 (3)				
T2	23 (12)	1.5 (0.4-5.2)	0.553	1.1 (0.3-4.4)	0.847
T3	102 (66)	2.7 (0.8-8.6)	0.995	1.0 (0.3-3.8)	0.948
T4	39 (31)	3.4 (1.0-11.2)	**0.043**	1.3 (0.3-5.1)	0.683
**N-stage**					
N0	67 (37)				
N1-N2	105 (75)	2.0 (1.3-2.9)	**0.001**	1.3 (0.8-2.0)	0.265
**Tumour size, continuous**	172 (112)	1.0 (1.0-1.0)	**0.010**	1.0 (1.0-1.0)	0.533
**Tumour differentiation**					
Well-moderate	73 (38)				
Poor	99 (74)	2.3 (1.5-3.3)	**<0.001**	1.9 (1.2-2.8)	**0.002**
**Tumour morphology**					
Intestinal type	63 (31)				
Pancreatobiliary type	109 (81)	2.3 (1.5-3.4)	**<0.001**	1.3 (0.7-2.3)	0.394
**Margins**					
R0	25 (7)				
R1-Rx	147 (105)	3.3 (1.5-7.0)	**0.002**	2.2 (1.0-4.9)	**0.046**
**Perineural growth**					
No	68 (33)				
Yes	104 (79)	2.4 (1.6-3.7)	**<0.001**	1.1 (0.6-1.8)	0.779
**Growth in lymphatic vessels**					
No	63 (30)				
Yes	109 (82)	2.2 (1.4-3.3)	**<0.001**	1.2 (0.8-1.9)	0.420
**Growth in blood vessels**					
No	131 (74)				
Yes	41 (38)	3.1 (2.1-4.7)	**<0.001**	2.4 (1.6-3.7)	**<0.001**
**Growth in peripancreatic fat**					
No	67 (30)				
Yes	105 (82)	2.8 (1.8-4.3)	**<0.001**	2.1 (1.4-3.3)	**0.001**

HR, hazard ratio. CI, confidence interval. Bold text indicates p < 0.05.

## Discussion

This is, to our best knowledge, the first report on standardized longitudinal opening and slicing of the common bile duct in the handling of PD-specimens with primary adenocarcinoma.

Our results confirm previous reports on standardized protocols in the pathology examination of operated periampullary adenocarcinomas by showing that a 1-mm cut-off in the assessment of margin status is relevant for overall survival, both in unadjusted analysis and after adjusting for other histopathology parameters. Microscopic re-evaluation of margin status revealed a larger proportion of involved margins than stated in the original reports. Thereby, the prognostic value of uninvolved margins was increased, regardless of other histopathology parameters. This suggests that a “guilty until proven innocent”-approach towards margins in pancreaticoduodenectomies gives more accurate prognostic information than the opposite approach. Moreover, survival in the large group of cases with unassessable margin status (Rx) differed significantly both from cases with uninvolved margins and from cases with involved margins, suggesting that it is not appropriate to classify these cases as R0.

The more frequent finding of growth in peripancreatic fat and perineural tumour growth in SP-cases compared to NSP-cases may be an effect of more extensive sampling in the periphery of the tumour as well as along the bile duct and margins in SP-cases compared with NSP-cases.

Tumour infiltration in blood vessels was more often found in NSP-cases than in SP-cases (29% vs 9%), which may be due to an unintended more thorough search for evaluable pathology parameters in SP-cases that had very little coverage on margins and lymph nodes. This model of explanation suggests that the proportion of cases with tumour infiltration in blood vessels in the NSP-group more accurately reflects the actual percentage of infiltration in blood vessels. As a cautionary remark, the possibility of a type I error, i.e. a false positive detection of significant differences between the NSP-group and the SP-group, should also be considered, since a large number of comparisons have been performed. A type II error, i.e. failure to detect the true incidence of involved blood vessels in the SP-group, is also possible due to the relatively small sample size in this group.

Comparisons of the incidence of involved margins between our SP-material, excluding duodenal origin, and other standardized series show 78% R1 (32/41) in our SP-group compared with 59% (32/54) and 61% (51/83) in the LEEPP-series [2,3]. The incidence of involved margins is often not comparable between SP-series and NSP-series, due to a 0-mm definition of margin involvement, or lack of definitions on margin involvement in NSP-series. The fraction of cases with involved lymph nodes is however comparable, showing that non-standardized series [4-10] report involved lymph nodes in less than 60% of cases, compared to more than 70% in our SP-group and in the LEEPP-series. If such differences are coincidental or actually statistically significant, as well as their potential clinical significance, remains unknown. In the present study, we were able to demonstrate a significantly higher number of involved lymph nodes in the specimens in the SP-group compared with the NSP-group, despite a temporal association between an increased number of lymph nodes harvested from the specimens by the surgeons and the studied standardized protocol.

In our material the differences in tumour origin between the SP-group and the NSP-group were significant. It is however not known if there are any clinically relevant differences between the tumour origins of standardized and non-standardized series. It has however previous been shown that the morphological distinction between intestinal and pancreatobiliary morphology has prognostic implications, not only in ampullary adenocarcinomas, but in all periampullary adenocarcinomas, regardless of tumour origin [14]. Moreover, while differences in the expression of cytokeratins and mucins according to morphology have been observed in ampullary carcinomas [15], these differences seem to be less evident in series stratified solely by the anatomical centre of the ampullary adenocarcinomas [16]. These findings suggest that morphological and molecular tumour characteristics have a greater prognostic impact than the appreciated tumour origin.

Despite a very different approach to the specimen, the results on tumour origin, N-stage and margin status in our standardized group are similar to the results of the LEEPP-series [2,3] and to a lesser degree similar to the results of two other variants on standardized protocols [17,18]. Whether or not our standardized protocol was more time consuming or more demanding than the LEEPP, and thus inferior due to practical reasons, has however not been studied.

## Conclusions

A 1-mm threshold for margin involvement is relevant for overall survival in operated periampullary adenocarcinomas, regardless of tumour origin and other histopathology parameters. Standardized protocols on sectioning of pancreaticoduodenectomy specimens seem to increase the yield of adverse prognostic histopathology parameters compared with non-standardized protocols. Standardizations in pancreatic pathology are needed to decrease unjustifiable variability in pathology reports, both for the sake of the treatment of individual patients and for the sake of future studies and clinical trials.

## Abbreviations

OS: Overall survival; HR: Hazard ratio; PD: Pancreaticoduodenectomy; LEEPP: Leeds pathology protocol; R1: Involved margins; Rx: Unknown margin status; R0: Uninvolved margins; N1-N2: Involved lymph nodes; SP: Standardized protocol; NSP: Non-standardized protocol; SMV: Superior mesenteric vein; SMA: Superior mesenteric artery; M: Median; IQR: Interquartile range; T-stage: Tumour stage; N-stage: Lymph node stage.

## Competing interests

The authors declare that they have no competing interests.

## Authors’ contributions

JE conceived of the study, collected data, performed the statistical analyses and drafted the manuscript. KJ participated in the design of the study, statistical analyses and drafting of the manuscript. Both authors read and approved the final manuscript.
